# Association of maternal body composition and diet on breast milk hormones and neonatal growth during the first month of lactation

**DOI:** 10.3389/fendo.2023.1090499

**Published:** 2023-03-02

**Authors:** David Ramiro-Cortijo, Pratibha Singh, Gloria Herranz Carrillo, Andrea Gila-Díaz, María A. Martín-Cabrejas, Camilia R. Martin, Silvia M. Arribas

**Affiliations:** ^1^ Department of Physiology, Faculty of Medicine, Universidad Autónoma de Madrid, Madrid, Spain; ^2^ Division of Gastroenterology, Beth Israel Deaconess Medical Center and Harvard Medical School, Boston, MA, United States; ^3^ Instituto Universitario de Estudios de la Mujer (IUEM), Universidad Autónoma de Madrid, Madrid, Spain; ^4^ Division of Neonatology, Hospital Clínico San Carlos, Instituto de Investigación Sanitaria del Hospital Clínico San Carlos (IdISSC), Madrid, Spain; ^5^ Department of Agricultural Chemistry and Food Science, Institute of Food Science Research (CIAL), Universidad Autónoma de Madrid, Consejo Superior de Investigación Científica (CSIC), Madrid, Spain; ^6^ Department of Neonatology, Beth Israel Deaconess Medical Center and Harvard Medical School, Boston, MA, United States; ^7^ Division of Translational Research, Beth Israel Deaconess Medical Center and Harvard Medical School, Boston, MA, United States

**Keywords:** hormones, breast milk, prematurity, growth, body composition, diet pattern

## Abstract

**Introduction:**

Preterm birth is associated with altered growth patterns and an increased risk of cardiometabolic diseases, with breast milk (BM) being a counteracting factor. Preterm infants also show alterations in adipokines and gut hormones influencing appetite and metabolism. Since these hormones are present in BM, it is possible that their levels may equilibrate deficiencies improving infant growth. We aimed to assess 1) the BM levels of ghrelin, resistin, leptin, insulin, peptide YY, and the gastrointestinal peptide in women with preterm and term labor; 2) the relationship between BM hormones and neonatal growth; and 3) the influence of maternal body composition and diet on these BM hormones.

**Methods:**

BM from 48 women (30 term and 18 preterm labor) was collected at days 7, 14, and 28 of lactation. Maternal body composition was evaluated by bioimpedance, and neonate anthropometric parameters were collected from medical records. The maternal dietary pattern was assessed by a 72-h dietary recall at days 7 and 28 of lactation. BM hormones were analyzed by the U-Plex Ultra-sensitive method. Data were analyzed using linear regression models. BM from women with preterm labor had lower ghrelin levels, with the other hormones being significantly higher compared to women with term delivery.

**Results:**

In premature infants, growth was positively associated with BM ghrelin, while, in term infants, it was positively associated with insulin and negatively with peptide YY. In the first week of lactation, women with preterm labor had higher body fat compared to women with term labor. In this group, ghrelin levels were positively associated with maternal body fat and with fiber and protein intake. In women with term labor, no associations between anthropometric parameters and BM hormones were found, and fiber intake was negatively associated with peptide YY.

**Discussion:**

Preterm labor is a factor influencing the levels of BM adipokines and gut hormones, with BM ghrelin being a relevant hormone for premature infant growth. Since ghrelin is lower in BM from women with preterm labor and the levels are associated with maternal fat storage and some dietary components, our data support the importance to monitor diet and body composition in women who gave birth prematurely to improve the BM hormonal status.

## 1 Introduction

The estimated global prevalence of preterm labor (pregnancy <37 completed weeks of gestation) has been rising from 9.6% in 2005 to 11% in 2020 (1, 2), mainly in low- and middle-income countries (3). Preterm infants have an increased risk of developing short-term comorbidities (4), as well as cardiometabolic diseases later in life (5). Premature birth also results in an altered growth pattern (6), with a slower rate in the short term (7) but with a faster growth later compared to term-born infants, a process called catch-up growth (8). At birth, premature infants also show alterations in the levels of adipokines and gut hormones that influence appetite and metabolism, such as leptin, adiponectin, and ghrelin, due to the inadequate adipose tissue storage and gastrointestinal maturation, which might be a risk factor for abnormal development and metabolic disorders later in life (9).

Breast milk (BM) is the gold standard for neonatal nutrition, providing not only macronutrients but also bioactive molecules. Metabolism- and appetite-regulating hormones have been identified in BM and may play a role in infant-feeding behavior, growth, and body composition (10–12). Furthermore, their presence in BM has been proposed to reduce the risk of cardiometabolic diseases (13, 14). The hormones regulating the food intake and energy homeostasis present in BM can be synthesized and excreted from the mammary gland or can be transported from maternal plasma (15). Insulin is synthetized by the pancreatic beta cells with a key role in glucose homeostasis control (16), with the maternal blood being the main source in BM (12). Leptin is an adipokine synthesized mainly by white adipose tissue (17), being a long-term anorexigenic hormone regulating body fat storage (18). Leptin can equally enter in the BM from maternal plasma and by the mammary gland synthesis (19, 20). Resistin is another adipokine present in BM (21), synthetized mainly by adipocytes and macrophages (22). The main role of resistin was described as an inflammatory mediator (22) antagonizing insulin actions (23). In addition to the above-mentioned hormones, some peptides from the gastrointestinal system that contribute to growth and appetite control are also present in BM. Peptide YY is an anorexigenic hormone produced by the gut and small intestine, identified in BM (24, 25), its main source remaining unclear. Ghrelin is a short-term orexigenic hormone, with a role in growth by inducing growth hormone secretion (26, 27). It is mainly synthesized by the stomach, although the pancreas, kidney, and placenta can produce it in modest amounts (28), being secreted in BM both by the mammary gland and maternal plasma (12, 29). The gastrointestinal peptide (GIP) is an incretin synthesized by gut after nutrient-induced signaling. In experimental animals, The GIP increases glucagon secretion, reduces appetite and, in the long-term, reduces body weight (30). BM GIP levels correlate with those in maternal plasma, suggesting that this is the main source (25).

Appetite hormones in BM are important factors not only for the neonatal appetite but also for neonatal growth regulation (24), especially in the first month of life. However, it is not clear if the levels of the appetite hormones in BM can affect growth, particularly in preterm infants. Another relevant aspect is the influence of maternal body composition and diet on BM hormone levels, mostly investigated in the context of maternal obesity and gestational diabetes (31, 32). Since BM hormone levels can influence infant growth (33), the relationship with the maternal nutritional status is important for counseling breastfeeding women. In this context, we hypothesize that BM appetite–regulating hormones influence neonatal growth differently in term and premature infants. In this study, we aimed to assess in the first month postpartum 1) differences in the BM levels of appetite-related hormones between women with term and preterm labor, 2) the role of these BM hormones on neonatal growth, and 3) the influence of maternal body composition and diet on their BM levels.

## 2 Material and methods

### 2.1 Study design and cohort

**Figure 1:**
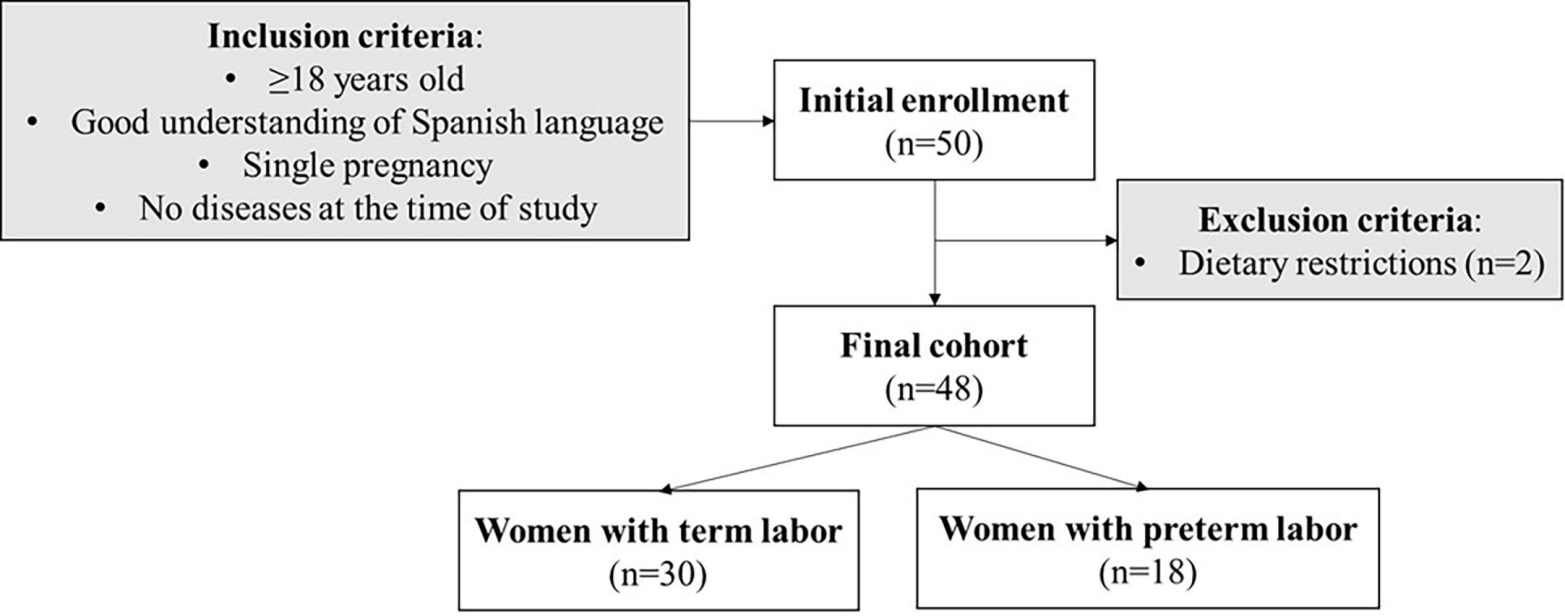
Flowchart of the women enrollment in the study and sample size (n) with the inclusion and exclusion criteria.

In this observational, longitudinal, and non-interventional study, the women were enrolled within the first 72 h postpartum at the Obstetrics and Gynecology and Neonatology Departments of Hospital Clínico San Carlos (HCSC, Madrid, Spain) from September 2019 to March 2020. The sample size was estimated considering that 11% of women would develop premature delivery, indicated by the prevalence of prematurity in 2020 (1, 2) and with an error margin of 5% and a statistical power of 80%. The estimated sample size would be 36 women. Considering this as an observational study, increasing the sample size versus the estimated could have benefits in suppressing the potential risk of bias. Maternal inclusion criteria were ≥18 years old, a good understanding of Spanish language, single pregnancy, and the absence of disease at the time of the study. Mothers with dietary restrictions (i.e., diet for competition sports, vegetarians, and vegans) were excluded from the study. The mothers who agreed to participate in the study signed informed consent. The final cohort included 48 women (**Figure 1**) who provided a BM sample at three time points, if possible (see below).

The present study was performed following the Declaration of Helsinki for studies on human subjects, and it was approved by the Ethical Committee of HCSC (Ref. 19/393-E).

### 2.2 Sociodemographic and gestational variables

Close to the enrollment day, the women filled a sociodemographic questionnaire including the maternal age (years), educational level (categorized as illiterate, middle school, high school, and university degree), employment situation (categorized as student, working, and unemployment), Spanish nationality (yes/no), and family size.

The obstetrical and labor clinical data were obtained from medical records and included the number of gestations (gravida) and previous abortions, use of assisted reproduction techniques (yes/no), vitamin D deficiency during this pregnancy (defined as 25-hydroxycholecalciferol blood levels <50 nmol/L in the second trimester), gestational hypothyroidism (defined as Thyroid Stimulating Hormone (TSH) blood levels >2.5 μU/ml in the first trimester and/or >3 μU/ml in the second and third trimesters), gestational anemia (hemoglobin levels <11 g/dl), gestational diabetes (defined as a positive result in the 100 g oral glucose tolerance test), preeclampsia (blood pressure >160/110 mmHg with proteinuria or thrombocytopenia after 20 weeks of gestation), use of antibiotic therapy during labor (yes/no), use of completed cycle of antenatal corticosteroids (yes/no), use of magnesium sulfate therapy (yes/no), type of labor (vaginal/C-section), and gestational age (weeks of gestation).

The neonate parameters were recorded from medical records and included the diagnoses of intrauterine growth restriction (defined as fetal growth <3rd percentile or <10th percentile with hemodynamic alterations), sex (male/female), Apgar score at 5 min, and Z-scores weight, length, and head circumference at birth considering to Fenton´s curves (34). The neonates were categorized as preterm (born <37 weeks of gestation) or term (born ≥37 weeks of gestation).

### 2.3 Maternal and neonatal anthropometric parameters

Maternal anthropometric parameters were measured at days 7 ± 1 and 28 ± 1 of lactation. Height (cm) was measured with a stadiometer (Seca 217, TAQ Sistemas Médicos, Madrid, Spain), and waist and hip circumferences (cm) were measured with an anthropometric tape with millimeter precision (Seca 201, TAQ Sistemas Médicos, Madrid, Spain). Body weight (kg), total body fat, and muscle mass (%) were assessed using a bioimpedance meter (Omron Healthcare HBF-514C Full Body Sensor W Scale, Madrid, Spain), according to the manufacturer’s instructions. From these parameters, the waist-to-hip index (waist/hip; WHI) and body mass index (BMI in kg/m^2^) were calculated.

The neonatal weight (g), length (cm), and head circumference (cm) were collected at 7, 14, and 28 days of life from medical records. From these data, the BMI was calculated following Olsen´s curves (35) and expressed in (g/cm^2^) × 10. Weight growth velocity was calculated as the exponential relationship between weight at birth and weight at the “j” point (W_j_) as a function of time, according to Patel´s model (36, 37). In this study, weight growth velocity was analyzed considering W_j_ as the discharge weight and expressed as (g/kg/day) using the described formula (38, 39). The length and head circumference growth velocities were calculated as a linear model and expressed in cm/day, as previously published (38).

### 2.4 Maternal 72-h dietary intake

Maternal dietary patterns were studied using the 72-h dietary recall (72hDR) questionnaire, a validated method to quantify an individual’s usual intake over a short period through open-ended questions (40–42). The 72hDR was obtained at days 7 ± 1 and 28 ± 1 of lactation. The women indicated the ingredients, food preparation methods, and quantities of everything they ingested during the three previous days, including any supplements and water intake. Women were instructed using the Spanish version of “Visual Guide to Food and Rations” (43) to obtain detailed information. The different foods and their ingredients were recorded manually and classified according to the meals. The composition of nutritional supplements was also included in the software. Thereafter, the analysis was carried out using DIAL software (version 3.11.9, Alce Ingeniería, Madrid, Spain) to calculate specific nutrient intake. The data provided information on energy (Kcal), carbohydrates (carbs.; g), protein (g), fat (g), saturated fatty acids (SFAs; g), monounsaturated fatty acids (MUFAs; g), polyunsaturated fatty acids (PUFAs; g), total cholesterol (mg), total dietary fiber (g), and water intake (ml). In addition, DIAL provided information regarding the intake of the following minerals: calcium (mg); iron (mg); iodine (mg); sodium (mg) and potassium (mg); and vitamins A (retinols, μg), B1 (thiamin; mg), B2 (riboflavin; mg), B3 (niacin; mg), B5 (pantothenic acid; mg), B6 (pyridoxines; mg), B9 (folic acids; μg), B12 (cobalamin; μg), biotin (μg), C (ascorbic acid; mg), D (μg), E (α-tocopherols; mg), and K (μg).

### 2.5 Breast milk collection and processing to obtain defatted phase

A 1 ml volume of BM was collected at 7 ± 2, 14 ± 2, and 28 ± 2 days of lactation, if available. It was not possible to get colostrum samples for ethical reasons due to the small volume that can be obtained, which are reserved for the neonate. BM was collected by each woman by hand self-expression with an electric breast pump (Symphony^®^ Medela, Barcelona, Spain). To collect the samples, the women washed their hands and cleaned their breast with a gauze with soap and water. BM was collected between 10:00 and 11:59 a.m., from both breasts (always after neonate feeding), pooled and immediately transferred to a glass bottle, and stored in a freezer. The time between extraction and processing took a maximum of 3 h. The sample was centrifuged three times (2,000 rpm for 5 min at 4°C) to obtain the defatted phase, minimizing turbidity. Glass serological pipettes were used to extract the aqueous layer, placed in a clean tube, and stored at −80°C until use. BM samples were analyzed within a month.

### 2.6 Breast milk hormone detection

A Human U-Plex Ultra-Sensitive Meso Scale Discovery (MSD) Kit (Meso Scale Diagnostics, LCC, Rockville, MD, USA) was used to determine in the BM-defatted phase insulin, leptin, ghrelin, the GIP, and peptide YY following the manufacture protocol. Briefly, each linker vial was conjugated with 200 μl of biotinylated antibody and incubated for 30 min at room temperature. Then, 200 μl of the stop solution was added in each linker vial and incubated for 30 min at room temperature. To prepare a multiplex coating solution, the linker-conjugated antibodies were mixed in a clean tube obtaining a total volume of 6 ml, and 50 μl volume of the multiplex coating solution was added to each well. The plate was covered and incubated shaking for 1 h at room temperature. Thereafter, the plate was washed three times with 150 μl of 0.05% Tween-20 Phosphate-Buffered Saline (PBS) 1X solution (v/v).

The standard curve was prepared according to manufacturer’s guidelines, and the BM-defatted samples were diluted 1.2-fold. A 50 μl volume of the standard curve or the diluted samples was added to the plate in duplicate. Thereafter, the plate was incubated under shaking for 2 h at room temperature. Then, the plate was washed three times with 150 μl of 0.05% Tween-20 PBS 1X solution (v/v). A 50 μl volume of the detection antibody was added to each well, and the plate was incubated under shaking for 1 h at room temperature. Thereafter, the plate was washed three times with 150 μl of 0.05% Tween-20 PBS 1X solution (v/v). Finally, 150 μl of the MSD-Gold read buffer reagent was added in each well. This is a highly sensitive method, in which the range and detection limits of the analyzed hormones were insulin = 0–736 pg/ml (0.32 pg/ml); leptin = 0–47,500 pg/ml (14.0 pg/ml); ghrelin = 0–2,710 pg/ml (1.70 pg/ml); GIP = 0–12,500 pg/ml (3.70 pg/ml); and peptide YY = 0–2,260 pg/ml (2.70 pg/ml).

The plate was read in the MESO QuickPlex SQ 120MM model 1300 system (Meso Scale Diagnostics, LCC, Rockville, MD, USA), and the data were extracted by the MSD discovery workbench analysis software. The hormones were reported in pg/ml, and the natural logarithmic transformation was used for the statistical analysis.

### 2.7 Breast milk resistin detection

A Single Spot R-Plex Human Assay (Meso Scale Diagnostics, LCC, Rockville, MD, USA) for resistin was also run. Resistin could not be included in the multiplex assay since some of the reagents for this analyte had a cross-competitive reaction with the reagents of the multiplex plate. Considering the manufacturer’s instructions, a 25 μl volume of biotinylated antibody was added to each well, and the plate was covered and incubated under shaking for 1 h at room temperature. Thereafter, the plate was washed three times with 150 μl of 0.05% Tween-20 PBS 1X solution (v/v). The standard curve was prepared according to the manufacturer’s guidelines, and the defatted BM samples were not diluted in this assay. A 25 μl volume of standard curve or BM-defatted samples were added to the plate in duplicate. The plate was incubated under shaking for 1 h at room temperature. Then, the plate was washed three times with 150 μl of 0.05% Tween-20 PBS 1X solution (v/v). A 50 μl volume of detection antibody was added to each well, and the plate was incubated under shaking for 1 h at room temperature. Thereafter, the plate was washed three times with 150 μl of 0.05% Tween-20 PBS 1X solution (v/v). Finally, a 150 μl volume of MSD-Gold read buffer reagent was added to each well. The resistin range was 0–2,500 pg/ml, and the low limit of detection was 0.13 pg/ml.

The plate was read in the MESO QuickPlex SQ 120MM model 1300 system (Meso Scale Diagnostics, LCC, Rockville, MD, USA), and the data were extracted by the MSD discovery workbench analysis software. Resistin was reported in pg/ml, and the natural logarithmic transformation was used for statistical analysis.

### 2.8 Statistical analysis

Statistical analysis was performed with R software within RStudio interface (version 2022.07.1 + 554, 2022, R Core Team, Vienna; Austria) using *rio, dplyr, compareGroups, ggpubr, devtools, stats, nlme, lme4*, and *ggplot2* packages.

Quantitative variables were expressed as the median and interquartile range [Q1; Q3], and qualitative variables were expressed as the relative frequency and sample size (n). In this study, we did not use methods to impute missing data. The probability (P) to show significance was established at value <5% in all analysis.

In the univariate analysis between term and preterm groups, the Mann–Whitney U test adjusted by Holm–Bonferroni multiple comparison was used for quantitative variables, and χ^2^ with Fisher correction was used for qualitative variables. In addition, Spearman-rho was used to assess correlations between the levels of BM hormones and maternal body composition. To analyze the neonatal growth curves and hormones over the lactation period, a two-way ANOVA was used to test the differences between groups (preterm/term), the day of lactation (d), and the interaction effect between the group and the day (g*d). In the growth curves, the interaction between the group and the sex was also reported.

In the multivariate analysis, two different models were used according to the strategy analysis to explore. Firstly, to explore the contribution of BM hormones to the neonatal growth pattern, linear regression models were used, with natural logarithmic transformation to normalize the weight, length, and head circumference. In this case, the prematurity and day subsets, adjusted by the neonatal sex and body fat of the women, was the strategy applied. Secondly, to detect if maternal body composition or dietary intakes were associated with BM hormones, the mixed models were used, considering woman by days as a random effect, and only those hormones showing association on neonatal growth were considered in this second analysis. In addition, the models were clustered by term or preterm infants and the anthropometry and dietary variables were normalized by natural logarithmic transformation. In all models, the coefficient (β) and standard error (SE) were reported.

## 3 Results

### 3.1 Maternal and neonatal characteristics at birth

There were no differences in sociodemographic characteristics between women with term and preterm labor. Regarding gestational variables, women with premature labor had higher rates of completed corticosteroid cycles and magnesium sulfate therapies than women with term labor. A higher proportion of male infants was observed in the preterm compared to the term group. No other differences in neonatal characteristics were detected (**Table 1**).

**Table 1:** Maternal and neonatal characteristics.

	Term (n=30)	Preterm (n=18)	P
Maternal age (years)	34.0 [32.2; 35.8]	35.0 [25.8; 39.5]	0.89
**Educational level**			0.26
High school	40.0% (12)	66.7% (12)	
University	43.3% (13)	27.8% (5)	
**Employment situation**			0.37
Student	0.0% (0)	5.6% (1)	
Working	63.3% (19)	55.6% (10)	
Unemployment	20.0% (6)	33.3% (6)	
**Nationality**			0.99
Spanish	56.7% (17)	61.1% (11)	
Non-Spanish	30.0% (9)	33.3% (6)	
Family size	4.0 [3.0; 4.0]	4.0 [3.0; 5.0]	0.98
Gravida	2.0 [2.0; 2.0]	2.0 [2.0; 2.0]	0.82
Abortion	0.0 [0.0; 0.8]	0.0 [0.0; 1.0]	0.25
Assisted reproduction techniques	3.3% (1)	0.0% (0)	0.99
Vitamin D deficiency	16.7% (5)	27.8% (5)	0.47
Gestational hypothyroidism	36.7% (11)	38.9% (7)	0.99
Gestational anemia	20.0% (6)	22.2% (4)	0.99
Gestational diabetes	23.3% (7)	16.7% (3)	0.72
Preeclampsia	6.7% (2)	16.7% (3)	0.36
Antibiotic therapy	33.3% (10)	50.0% (9)	0.42
Corticosteroids (completed cycle)	0.0% (0)	61.1% (11)	<0.001
Magnesium sulfate therapy	0.0% (0)	50.0% (9)	<0.001
C-section	26.7% (8)	38.9% (7)	0.57
Gestational age (weeks)	38.9 [38.0; 39.9]	28.7 [27.2; 34.0]	<0.001
Neonate sex (male)	26.7% (8)	61.1% (11)	0.040
Apgar 5 min	10.0 [9.0; 10.0]	9.0 [8.0; 10.0]	0.11
Birth weight (Z-score)	-0.03 [-0.62; 0.32]	0.12 [-0.84; 0.80]	0.54
Birth length (Z-score)	-0.32 [-0.89; 0.17]	0.14 [-0.60; 0.92]	0.16
Birth head circumference (Z-score)	-0.04 [-0.35; 0.50]	-0.22 [-0.67; 0.26]	0.41
Intrauterine growth restriction	10.0% (3)	0.0% (0)	0.54

Data show the median and interquartile range [Q1; Q3] for quantitative variables and relative frequency and the sample size (n) for qualitative variables. The P-value (P) was extracted by the Mann–Whitney U test adjusted by Holm–Bonferroni multiple comparison in quantitative variables or χ^2^ in qualitative variables.

### 3.2 Neonatal growth during the first month of life

**Figure 2:**
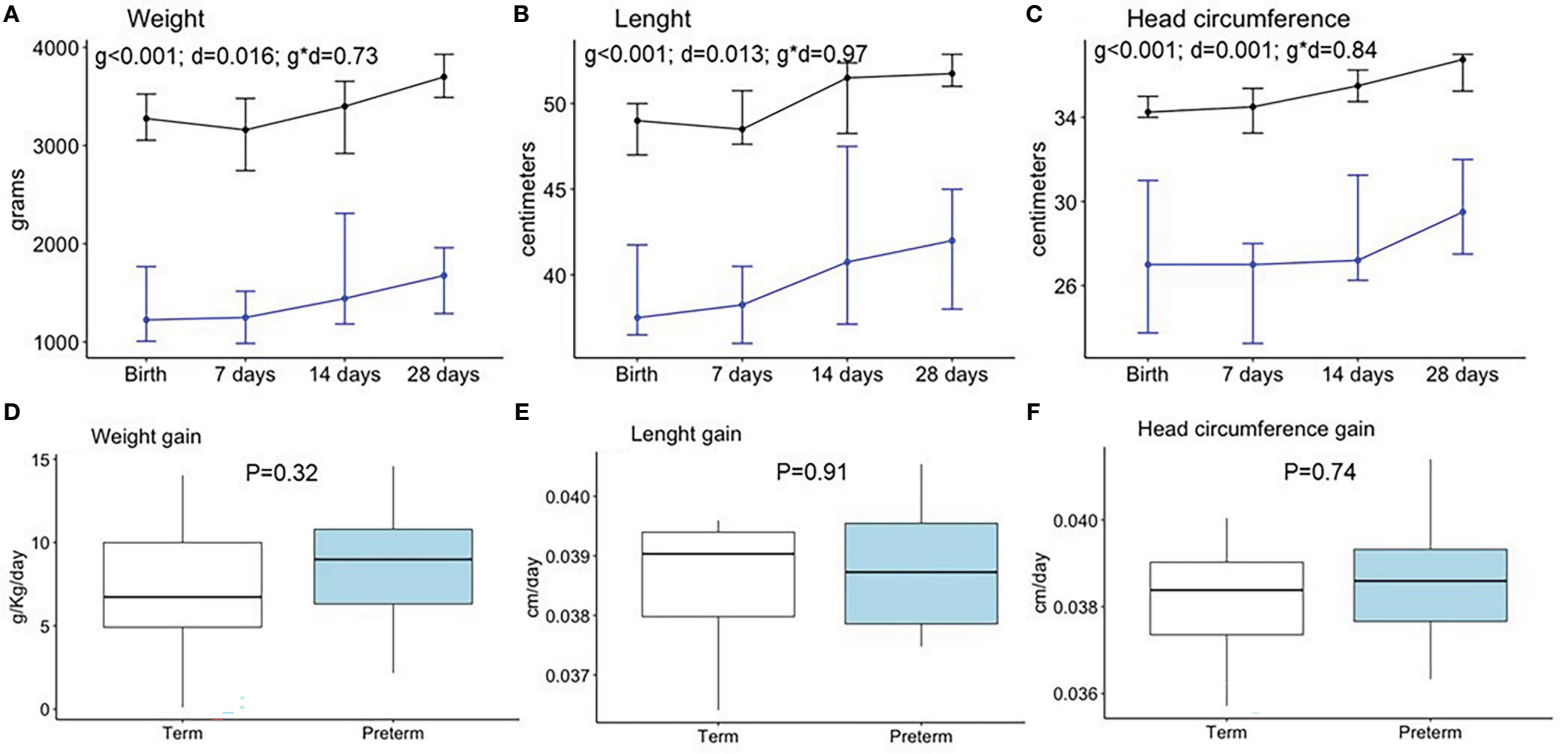
Neonatal anthropometry pattern during the first month of life in weight **(A)**, length **(B)**, and head circumference **(C)** and weight gain **(D)**, length gain **(E)**, and head circumference gain **(F)**. Data show the median and interquartile range [Q1; Q3]. Black lines show term infants (Birth n=30, 7 days n=22, 14 days n=17, 28 days n=15), and blue lines show preterm infants (Birth n=18, 7 days n=15, 14 days n=13, 28 days n=12). In the two way-ANOVA, the following were considered as factors: preterm/term (group = g), birth/7days/14 days/28 days (day = d), and interaction effect group and day (g * d). The P-value (P) was extracted by the Mann–Whitney U test in the boxplots.

We analyzed the growth pattern and velocity during the first month of life. The weight, length, BMI, and head circumference were significantly larger in term compared to preterm neonates at all time points (**Figures 2A-C**, **S1A**). However, growth velocities were not significantly different between groups (**Figures 2D-F**, **S1B**). The prematurity and days did not show interaction in any of the neonatal anthropometry parameters. No interactive effect was observed between prematurity and sex on growth (data not shown).

### 3.3 Breast milk hormone levels in the first month of lactation

**Figure 3:**
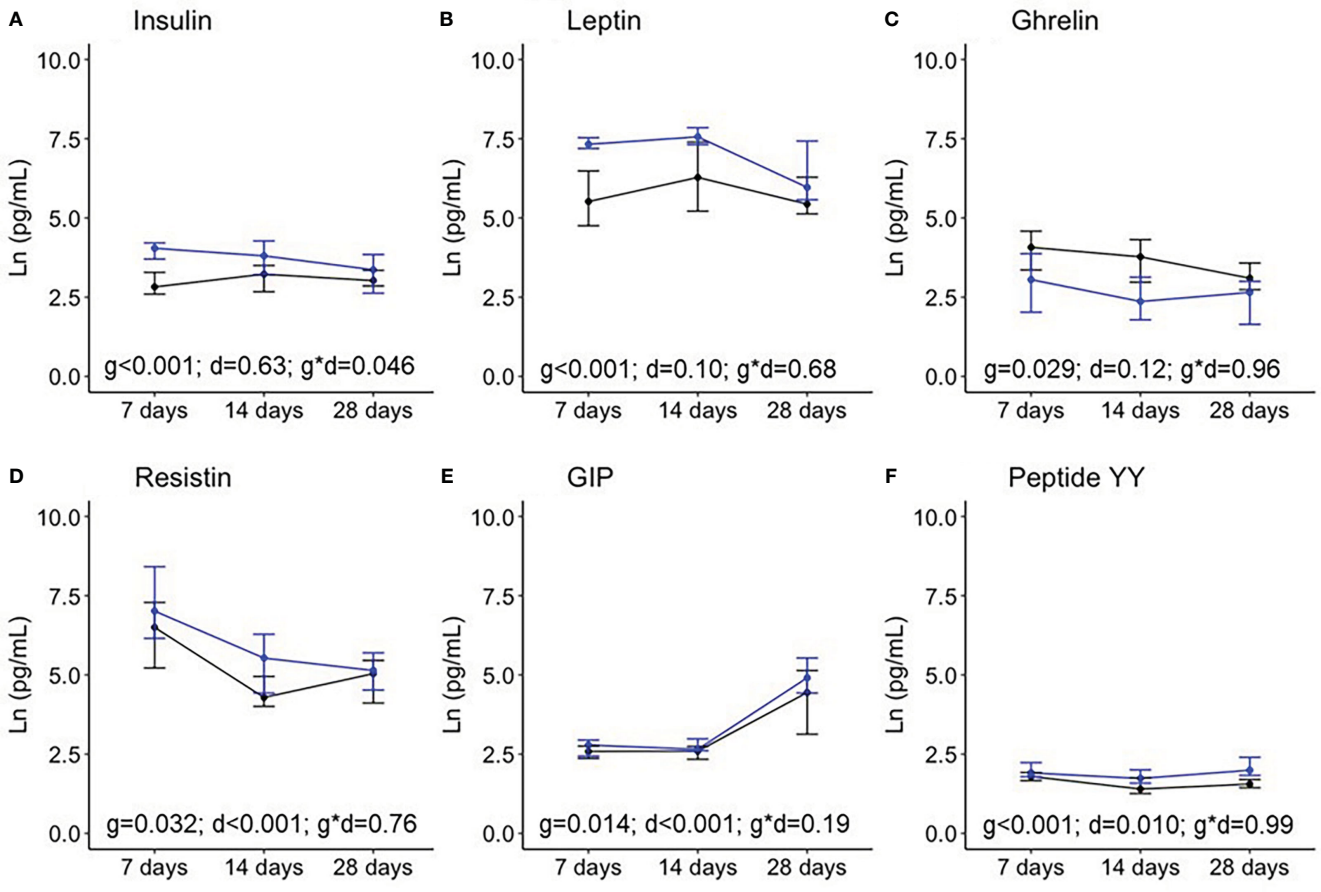
Breast milk levels of insulin **(A)**, leptin **(B)**, ghrelin **(C)**, resistin **(D)**, the gastrointestinal peptide (GIP); **(E)**, and peptide YY **(F)** during the first month of lactation. Data show the median and interquartile range [Q1; Q3]. Black lines show term infants (7 days n = 20, 14 days n = 18, 28 days n = 17), and blue lines show preterm infants (7 days n = 13, 14 days n = 10, 28 days n = 10). In the two way-ANOVA, the following were considered as factors: preterm/term (group = g), birth/7days/14 days/28 days (day = d), and the interaction effect group and day (g * d).

**Figure 3** shows the levels of the hormones analyzed at the three time points during the first month of lactation. The “Birth” point was excluded from the analysis since BM was not obtained in the hours immediately after birth. When compared between two groups, the levels of insulin, leptin, resistin, the GIP, and peptide YY were significantly higher in BM from women with preterm compared with term labor, while ghrelin was significantly lower.

With respect to variation over time along the first month of lactation, the BM levels of resistin significantly decreased, while GIP and peptide YY increased, with insulin, leptin, and ghrelin being stable levels. Only insulin levels showed an interaction effect between prematurity and days, indicating that, in BM from women with premature labor, insulin decreases during the first weeks postpartum, while it is stable in BM from mothers with term labor (**Figure 3**).

### 3.4 Association between breast milk hormones and neonatal growth

To explore the contribution of BM hormones to the neonatal growth pattern, linear regression models were used adjusted by the sex and women fat mass percentage, preterm, and day in separated sets.

**Figure 4:**
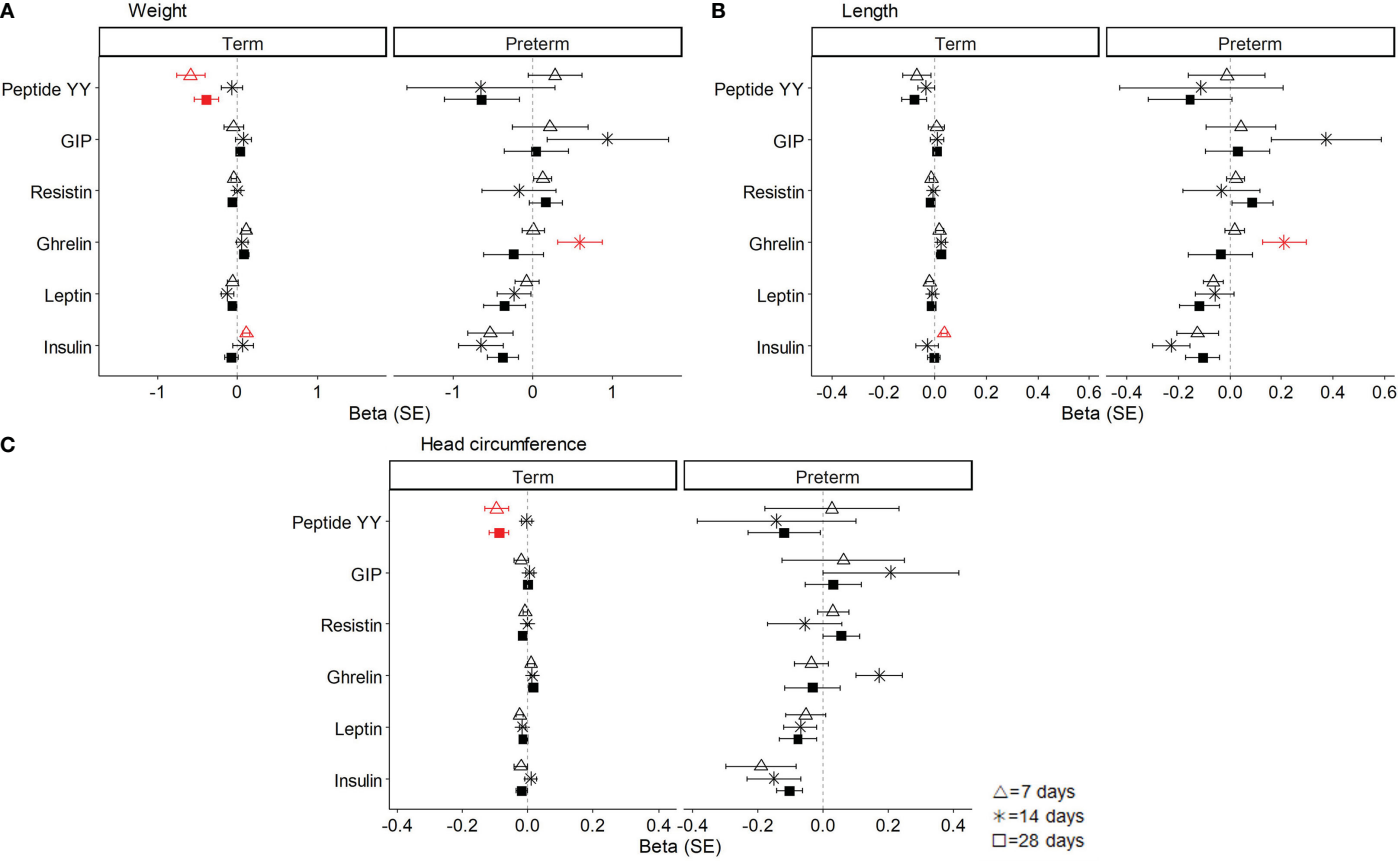
Linear regression models showing the association between breast milk (BM) hormones and neonatal weight **(A)**, length **(B)**, and head circumference **(C)**. The neonatal growth variables were normalized by logarithmic transformation, and the coefficients (beta) for each day of lactation were separately analyzed. Data show beta and standard error (SE). The red lines mean significant association (P < 0.05), and non-significant associations are represented in black. All models were adjusted by sex and women fat mass percentage. GIP, gastrointestinal peptide.

In term neonates, weight gain along lactation was associated with a decrease in BM peptide YY at 7 and 28 days of lactation and with an increase in insulin at day 7. In preterm neonates, weight gain was associated with the increase in BM ghrelin at day 14 (**Figure 4A**). In term neonates, length gain was associated with the increase in BM insulin levels at day 7. In preterm neonates, length gain was associated with the increase in BM ghrelin at day 14 (**Figure 4B**). In term neonates, the increase in head circumference was associated with a decrease in BM peptide YY at day 7 and 28 of lactation. In preterm neonates, there was no association between head circumference and BM hormones (**Figure 4C**).

### 3.5 Maternal body composition and dietary pattern during the first month of lactation

At 7 days, women with premature labor had significantly higher BMI and body fat and lower muscle percentage than women with term labor. However, at day 28, no statistical differences were detected between the groups in any of the anthropometric parameters (**Table 2**).

**Table 2:** Maternal body composition and dietary intakes between groups in the first month of lactation.

	7 days			28 days		
Body composition	Term (n=21)	Preterm (n=16)	P	Term (n=18)	Preterm (n=11)	P
BMI (kg/m^2^)	24.7 [23.2; 27.5]	28.8 [26.1; 30.6]	0.036	24.9 [23.1; 28.3]	28.7 [25.0; 30.9]	0.09
WHI	0.90 [0.85; 0.91]	0.90 [0.84; 0.94]	0.90	0.87 [0.81; 0.89]	0.88 [0.84; 0.93]	0.65
Body fat mass (%)	35.9 [32.1; 41.6]	42.4 [37.4; 46.2]	0.021	38.5 [35.6; 42.0]	40.8 [37.8; 46.9]	0.11
Muscle mass (%)	26.1 [25.6; 28.9]	24.6 [22.8; 26.4]	0.033	25.8 [24.6; 27.3]	25.9 [22.9; 26.4]	0.31
**Dietary intakes**	**Term** **(n=20)**	**Preterm (n=12)**	**P**	**Term** **(n=14)**	**Preterm (n=6)**	**P**
Energy (Kcal)	2,002 [1,399; 2,400]	2,109 [1,882; 2,432]	0.41	2,150 [1,785; 2,248]	1,820 [1,528; 1,878]	0.28
Proteins (g)	95.6 [72.4; 106]	87.6 [78.0; 100]	0.76	90.9 [85.0; 106]	90.0 [79.9; 99.8]	0.54
Carbohydrates (g)	204 [168; 216]	204 [171; 268]	0.71	194 [162; 214]	156 [144; 186]	0.23
Fat (g)	95.5 [54.6; 114]	89.4 [77.2; 108]	0.88	100 [73.6; 105]	76.2 [58.6; 90.9]	0.14
SFAs (g)	31.2 [21.6; 40.2]	31.0 [22.6; 34.0]	0.92	30.6 [22.7; 38.4]	26.2 [21.4; 32.9]	0.41
MUFAs (g)	42.2 [21.7; 47.3]	39.6 [33.7; 45.0]	0.82	40.5 [32.5; 46.3]	30.1 [21.3; 41.6]	0.14
PUFAs (g)	11.7 [5.40; 14.9]	12.6 [10.9; 14.0]	0.37	14.2 [12.5; 16.1]	11.6 [8.50; 12.6]	0.026
Cholesterol (mg)	418 [271; 464]	356 [312; 361]	0.28	378 [308; 462]	311 [227; 394]	0.30
Fiber (g)	19.3 [13.2; 24.5]	22.1 [19.7; 28.0]	0.44	21.8 [15.4; 27.8]	17.3 [15.9; 21.1]	0.74
Water (ml)	1,564 [1,216; 2,362]	1,451 [1,349; 2,272]	0.97	1,901 [1,359; 2,868]	1,654 [1,261; 2,776]	0.81

Data show median and interquartile range [Q1; Q3]. The P-value (P) was extracted by the Mann–Whitney U test adjusted by Holm–Bonferroni multiple comparison. BMI, body mass index; WHI, waist-to-hip index; met., metabolic; SFAs, saturated fatty acids; MUFAs, monounsaturated fatty acids; PUFAs, polyunsaturated fatty acids.

At day 7, the dietary pattern was not statistically different between women with or without term labor, neither in macronutrients nor in mineral or vitamin intakes. However, at day 28, the women with premature labor significantly decreased their PUFAs and vitamin E intake compared to women with term labor (**Tables 2**, **S1**). We had some missing data of the 72hDR questionnaires by day 28, due to the lack of answers by the participant, or the change of residence, among other reasons. Therefore, to assess the possible interference of these follow-up loses with the results, we performed a secondary intention to treat analysis comparing those women who only answered the 72hDR at day 7 with women who finished the study. This analysis showed that, in women with term delivery, those who did not complete the study had a lower PUFA intake (5.40 [4.70; 8.95] g) compared to those who completed the study (14.3 [12.1; 15.6] g; P=0.004). However, in women with preterm delivery, those who did not complete the study had similar PUFA intake (12.9 [10.8; 14.9] g) compared to those who completed the study (12.5 [12.2; 12.7] g; P=0.57).

### 3.6 Association between maternal body composition and dietary intake with breast milk hormones

**Figure 5:**
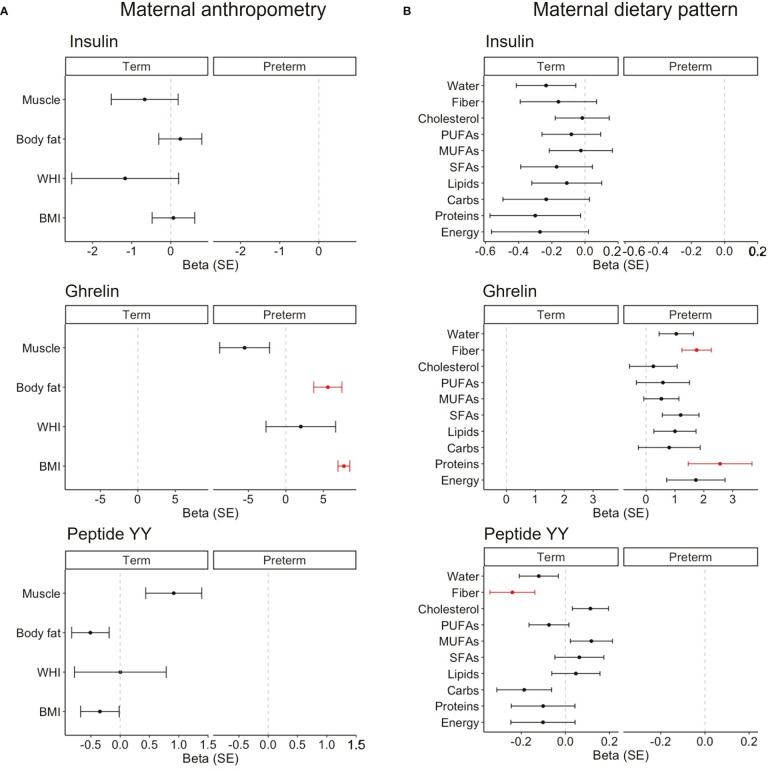
Linear mixed models on the association between maternal anthropometry **(A)** and the maternal dietary pattern **(B)** on BM hormones that influenced neonatal growth in each cohort. Women by day were included as the random effect, and maternal variables were normalized by logarithmic transformation. Data show beta and SE. Red lines mean significant association (P < 0.05), and non-significant associations are represented in black. BMI, body mass index; WHI, waist-to-hip index; SFAs, saturated fatty acids; MUFAs, monounsaturated fatty acids; PUFAs, polyunsaturated fatty acids.

The mixed models, considering woman by days as a random effect, were built to study if maternal body composition (**Figure 5A**) or dietary intake (**Figure 5B**) were associated with BM hormones. Only the hormones that influenced the neonatal growth were considered in the analysis. In women with term labor, none of the anthropometric variables were associated with BM hormones. However, in women with premature labor, the BMI and body fat mass were positively associated with ghrelin levels in their BM (**Figure 5A**). This means that for each increased unit in the BMI or body fat mass, ghrelin levels also increased by 7.7 ± 0.8 and 5.6 ± 1.9 units, respectively. We also performed a correlation analysis across gestational ages to evaluate the influence of maternal body composition on BM hormones. We found a positive correlation between the BMI and body fat with insulin and leptin levels; the waist–hip index was also positively correlated with leptin, and body fat was positively correlated with the GIP. However, we did not detect correlations between any of the maternal body composition parameters and BM ghrelin across gestational ages (**Figure S2**). In addition, we analyzed the possible influences of maternal body composition on those BM hormones that did not influence neonatal growth. We only found a negative association between the percentage of muscle with BM insulin levels in women with premature labor (**Figure S3A**).

Regarding the dietary pattern, in women with term labor, we did not detect any association with insulin levels. However, fiber intake was negatively associated with peptide BM YY (β = -0.2 ± 0.1; **Figure 5B**). In women with preterm labor, protein and fiber intake were positively associated with the levels of BM ghrelin (β = 2.6 ± 1.1 and β = 1.8 ± 0.5, respectively; **Figure 5B**). In addition, we analyzed the possible influences of the maternal diet on those BM hormones that did not influence neonatal growth. We only found a positive association between cholesterol intake with BM ghrelin levels in women with term labor (**Figure S3B**).

A secondary analysis in the preterm neonatal cohort demonstrated that the use of antenatal corticosteroids had a negative impact on insulin, leptin, ghrelin, resistin, and peptide YY levels for infant weight. Regarding length, a negative association was found with leptin, ghrelin, and resistin, and no association was observed with head circumference. On the other hand, the use of antenatal magnesium sulfate had a negative association on the GIP and peptide YY for head circumference (**Table S2**).

## 4 Discussion

**Figure 6:**
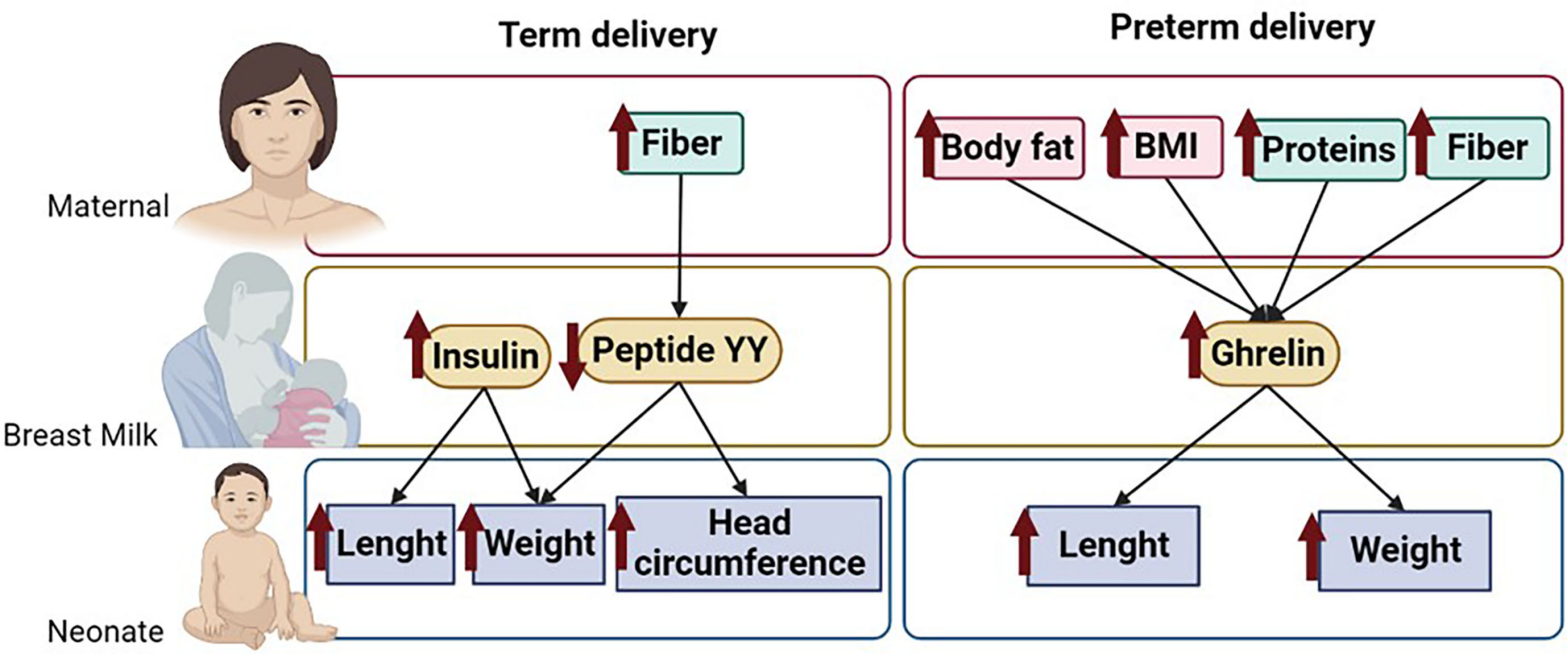
Summary of the main results of the study. Figure shows the maternal dietary components and body composition parameters that influence BM hormone levels and their association on neonatal growth. The arrows indicate associations between parameters. .

This article analyzes the levels of some BM hormones that influence appetite and metabolism comparing mothers with term and preterm delivery, focused on their association with the growth of their neonates during the first month of lactation. We also evaluate the influence of the maternal nutritional status on these BM hormones. We found important variations in the levels of the analyzed hormones between term and preterm delivery and a differential influence of these hormones on infant growth depending on the type of labor. In term infants, growth was positively affected by BM insulin levels, while peptide YY exerted a negative impact. On the other hand, in preterm infants, growth was mainly influenced by BM ghrelin levels, which had a positive association on body weight and length gain. Regarding the influence of maternal factors on BM hormone levels, our data indicate that, in women with term labor, body composition does not seem to affect BM hormones. However, in women who gave birth prematurely, fat mass was positively associated with BM ghrelin levels. With respect to the influence of diet, we detected that fiber intake affected some of the BM hormones which influenced neonatal growth, being negatively associated with the levels of peptide YY in women with term delivery and positively associated with ghrelin in women with preterm labor (summary shown in **Figure 6**). The present study demonstrates that preterm labor affects the content of appetite hormones in BM, being influenced by maternal body fat and some dietary components. Since these hormones exert an influence on infant growth, it would be important to monitor maternal nutrition and body composition during lactation.

BM is the gold standard for infant nutrition, and gaining knowledge in its components is critical to understand how it influences infant growth and development. This is particularly relevant in the population of preterm neonates, which exhibit a growth deficiency in the short term (7), followed by growth acceleration, which may contribute to the observed high metabolic risk of this population (9). In this context, BM hormones that modulate metabolism and appetite are relevant since they may contribute to counteract these growth alterations and explain the reduction of risk to develop obesity and diabetes in individuals who were breastfeed (13, 14). Even though BM is the best food for the neonate, in some situations, it is not possible to breastfeed, and, therefore, the knowledge gained about BM hormones can guide the pharmaceutical industry to develop a better infant formula.

### 4.1 Differences in BM hormones between term and preterm delivery

Our first objective was to evaluate possible differences in hormones that influence appetite and metabolism in BM from women with term and preterm labor. We found significant differences in the levels of insulin, leptin, resistin, ghrelin, the GIP, and peptide YY between these groups, all being higher in women with preterm labor, except for ghrelin, which showed the opposite trend. The studied hormones can access BM from the maternal plasma or can be produced by lactocytes (15). Maternal circulation seems to be the main source of insulin (12), due to the limited capacity of mammary epithelial cells to synthesize it (20), and of resistin, since its levels in BM correlate with those found in maternal plasma (21). On the other hand, the mammary gland and maternal plasma contribute equally to BM ghrelin levels (12) and leptin can enter BM from maternal plasma (44) or can be synthesized by lactocytes (20). Considering that the mammary gland has an ongoing development during the whole pregnancy (45), women with preterm delivery have an underdeveloped gland, with leaky tight junctions (46). This may account, at least in part, for the observed differences, by modifying the passage of molecules from serum or the capacity of lactocyte synthesis. We did not analyze the plasma levels of the studied hormones, and, therefore, we cannot determine if they account for the differences observed between groups. This is a limitation of our study, and future work measuring hormone levels simultaneously in BM and in maternal plasma will allow to determine the relative contribution of lactocyte synthesis and passage from the blood. In addition, it is possible that, in the present study, the levels of some hormones may be underestimated since we used defatted BM and it has been shown that ghrelin (29) and leptin levels are positively correlated with BM fat content (20). However, the differences between term and preterm BM are still valid since previous meta-analysis demonstrated similar fat content in the BM of women with term and preterm labor (47). Overall, our study demonstrates that prematurity is a factor contributing to changes in the BM levels of relevant hormones that modulate appetite and infant growth.

### 4.2 Influence of maternal nutrition and body composition on breast milk hormones

Maternal body composition and the dietary pattern have been previously shown to influence BM hormones. For example, a positive association between maternal BMI and BM leptin concentration is consistently found in most studies (24, 48, 49) and was also confirmed in our cohort, together with a positive correlation between the BMI, fat mass, and insulin levels. However, the evidence for an association between maternal body composition and other hormones in BM is lacking (48). Our study also evidenced that ghrelin concentration was positively associated with maternal fat mass but only in BM from women with preterm labor. Higher fat mass was observed in this group (likely related to the interruption of pregnancy due to preterm labor, when fat accumulation is high), normalizing at the end of the first month. Moreover, there is evidence that ghrelin synthesis peaks around mid-pregnancy in association with an increase in maternal weight gain and fat storage during gestation (50). In addition, it is possible that women with preterm labor accumulated more body fat due to the dysregulation of their appetite. Prematurity is a key factor for maternal psychological stress (51, 52), and exposure to a stressor has been shown to increase ghrelin levels in humans, which is considered a relevant hormone for stress-induced hyperphagia (53). Therefore, it is possible that women with preterm labor, particularly those with higher body fat, may also have elevated plasma ghrelin, which would be interesting to confirm in future studies. Nevertheless, we found that BM ghrelin levels were lower in women with preterm delivery, compared to those with term labor. Since BM ghrelin is both released from maternal plasma and from the mammary gland (29), we suggest that the lower BM content in women with preterm labor is due to lactocyte immaturity (54).

We also analyzed the impact of the maternal diet on BM hormone levels. Overall, the diet between women with term or preterm delivery did not show marked differences. We only detected differences in PUFA intake at day 28, being lower in women with preterm delivery. This may be related to a worse dietary behavior in this group, as we have previously demonstrated (55). However, we must consider a possible overestimation of PUFA intake in the women with term delivery due to the dropout of those with lower intake, as shown by the intention-to-treat analysis. With this consideration, it is necessary to control the potential high risk of significance bias. An interesting observation was the interaction between fiber intake and the levels of some BM hormones. In women with term delivery, fiber intake was negatively associated with BM peptide YY levels, while in women with premature labor, it was positively associated with BM ghrelin levels. This is a surprising result since ghrelin is an orexigenic hormone, which would be most likely decreased by fiber ingestion since it promotes satiation (56). However, it has been demonstrated that different types of dietary fiber have different effects on satiety and the plasma levels of gut hormones, such as ghrelin and peptide YY (57). Furthermore, the influence of fiber intake on gut satiety–related hormone release markedly differs depending on the population studied, their glucose homeostasis, and the chemical nature of the fiber (soluble versus insoluble) (58, 59). Given the fact that ghrelin in BM comes from plasma and the mammary gland (29), further analysis assessing its level in maternal blood would shed light on the observed relationship between maternal fiber intake and ghrelin levels in BM.

### 4.3 Influence of breast milk hormones on infant growth

Our third, and most important, aim was to assess the association of appetite-related BM hormone levels on infant growth. As expected, during the first month of lactation, the body weight, length, and head circumference were larger in term neonates. However, we did not find differences in neonatal Z-scores or growth velocities between term and preterm neonates. It has been proposed that preterm infants exhibit growth acceleration, being slower in those fed with BM (8). However, we did not observe catch-up growth in the present study, which may be related to the fact that this growth acceleration is influenced by several factors, such as intrauterine growth restriction, birth weight, or comorbidities, among others (60), and may not take place until the pathological factor disappears, usually after the first year of life (61).

We observed that in term and preterm neonates, growth was influenced by different hormones. In term neonates, weight and length were positively associated with BM insulin levels. It has been proposed that BM insulin plays a role in both neonatal growth in the first month of life (62) and at 1 year of age (63), and high systemic insulin levels have been associated with larger growth (64). However, it has been put forward that insulin BM levels and infant growth have a U-shape pattern and that intermediate concentrations of insulin in BM may be optimal to support infant metabolism, while insufficient or excessive insulin may impair this process (63). In term infants, we also found that BM peptide YY levels were negatively associated with weight and head circumference during the first month of life. Since peptide YY is an anorexigenic signal, it is possible that BM content reduces appetite and thus energy intake. In preterm infants, growth was associated with a different pattern of hormones, the most relevant being ghrelin, which showed a positive correlation with growth. Our findings agree with the correlation between serum ghrelin and skinfold size observed in breastfed infants (65). The growth-promoting effects of ghrelin could be related to its orexigenic actions, which may increase appetite in the neonate. Another possible effect is through the release of growth hormones (66). Our observation of higher BM ghrelin levels in women with high body fat suggest that, in women with preterm labor, higher fat accumulation would be beneficial to increase this orexigenic hormone in their BM with a beneficial effect for premature infant growth. Our data showing the modulation of infant growth by BM hormones indicate that they must reach the plasma and, therefore, they must be absorbed. There is evidence that infants can enterally absorb leptin, including preterm neonates, shown by the correlation between leptin in BM and in infant plasma (67). Leptin receptors have been located in human intestinal mucosae, even since the fetal stage (68), and, in rats, it has been demonstrated that oral leptin is absorbed by the neonatal epithelium, exerting biological effects (69). Regarding how peptide hormones can reach the intestinal mucosae without degradation, it is possible that the lower pepsin secretion and the higher gastric pH in neonates hydrolyze less protein in the infant’s stomach (70, 71). Given the substantial amount of evidence showing the role of BM hormones in neonatal growth, this aspect needs further attention. In addition to the effect of BM hormones, we cannot rule out the contribution of infant endogenous hormones for growth. However, it must be considered that, at birth, the levels of many of these hormones are altered in preterm infants (9), and the dysregulation in adipokines and gut-derived hormones may negatively contribute to immediate postnatal growth or program the development of metabolic disease later in life. Therefore, their source from maternal BM may have a regulatory effect, equilibrating levels. In our study, we must also take into account the fact that, in most cases, preterm infants were fed with supplements apart of BM, and we did not assess the relative amount of BM ingested, which is a limitation of our study. The use of supplements for preterm infants could be beneficial for growth if BM intake is low. However, if the mother can provide sufficient volume, attention should be focused on supporting maternal nutrition to improve BM macronutrients and bioactive molecules, such as hormones, which would, in turn, benefit infant growth.

We also analyzed the impact of antenatal corticosteroids and magnesium sulfate, which are commonly used in the context of prematurity, and there is controversy regarding some of their effects (72). We found a negative influence of corticosteroid administration on infant weight and length through alterations in BM hormone levels. To the best of our knowledge, there is no information about the effect of antenatal corticosteroid treatment on BM hormone secretion, but it is known that they reduce the volume of BM (73). This has also been confirmed in experimental animals, also showing that, together with reduced BM yield, neonatal growth is compromised (74). Our data point in this direction, with a negative association of the use of antenatal corticoids and magnesium sulfate on neonatal growth through the BM hormones. This aspect deserves further studies.

## 5 Conclusions

In conclusion, we found important variations in the content of BM hormones between term and preterm delivery, which may be related to different mammary gland development and maternal body composition. However, it must be considered that, in addition to preterm labor itself, other maternal influences, such as pregnancy complications or a woman’s genetic background, may also influence BM hormones. Our study also evidences that BM insulin is the main growth-driving hormone in term neonates, while ghrelin is relevant in premature infants. Given the fact that ghrelin is lower in preterm BM, being associated with maternal fat storage and maternal dietary components, our data support the importance to monitor diet and body composition in women who gave birth prematurely to improve the BM hormonal status. Our data also support a negative influence of corticosteroid administration on infant growth through alterations in BM hormone levels.

## Data availability statement

The raw data supporting the conclusions of this article will be made available by the authors, without undue reservation.

## Ethics statement

The studies involving human participants were reviewed and approved by Ethical Committee of Hospital Clínico San Carlos (Ref. 19/393-E). Written informed consent to participate in this study was provided by the participants’ legal guardian/next of kin.

## Author contributions

DR-C has contributed to the design and acquisition; performed the analysis and interpretation of data; and drafted the manuscript, tables, and figures. PS has contributed to the acquisition and interpretation of data and drafted the manuscript. GC and AG-D have contributed to the recruitment of infants and collection of samples. MM-C and CM have contributed to the interpretation of data and editing of the manuscript. SA has contributed to the conception, design, interpretation, and review of the manuscript. All authors contributed to the article and approved the submitted version.
