# Supplementary material for: Association of maternal body composition and diet on breast milk hormones and neonatal growth during the first month of lactation
Source: Front Endocrinol (Lausanne). 2023 Mar 2;14:1090499. doi: 10.3389/fendo.2023.1090499 (PMC10018215; doi:10.3389/fendo.2023.1090499)

Supplementary Material

Figure S1. Neonatal body mass index (BMI) pattern (A) and its change (B) during the first month of life in term and preterm neonates. Data show median and interquartile range [Q1; Q3]. Analysis was performed by two way-ANOVA considering as factors: preterm/term (group=g), birth/7days/14 days/28 days (day=d), and interaction effect group and day (g*d). The P-value (P) was extracted by Mann-Whitney U test, and it is shown in the boxplot. Term = 30 and preterm = 18.

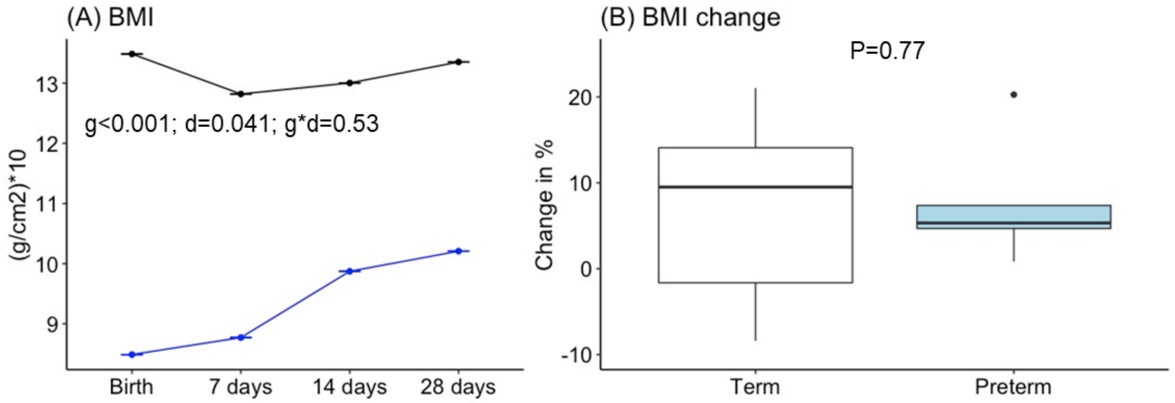

Table S1. Maternal mineral and vitamin intake in women with term and preterm labor in the first month of lactation. Data show median and interquartile range [Q1; Q3]. The P-value (P) was extracted by Mann-Whitney U test adjusted by Holm-Bonferroni multiple comparison. Vit.: Vitamin.

	**7 days**			**28 days**		
**Dietary intake**	**Term (n=20)**	**Preterm (n=12)**	**P**	**Term (n=14)**	**Preterm (n=6)**	**P**
Calcium (mg)	1028 [803; 1241]	771 [716; 1062]	0.13	1094 [868; 1308]	918 [716; 1125]	0.25
Iron (mg)	18.0 [13.5; 35.6]	17.0 [13.3; 57.1]	0.88	26.6 [18.8; 88.6]	28.8 [16.5; 76.3]	0.81
Iodin (mg)	143 [74.3; 286]	98.6 [70.7; 148]	0.41	296 [195; 312]	172 [98.8; 282]	0.41
Sodium (mg)	2142 [1506; 2613]	1824 [1506; 2213]	0.33	1852 [1733; 2406]	1867 [1727; 2056]	0.36
Potassium (mg)	3074 [2592; 3318]	3029 [2794; 3408]	0.76	3307 [2404; 3811]	2812 [2673; 4284]	0.68
Vit. B1 (mg)	1.70 [1.28; 2.25]	1.35 [1.20; 2.05]	0.47	2.40 [1.62; 2.58]	1.30 [0.92; 1.48]	0.10
Vit. B2 (mg)	2.30 [1.70; 3.35]	1.80 [1.20; 2.30]	0.19	3.25 [2.25; 3.48]	2.00 [1.35; 2.50]	0.08
Vit. B3 (mg)	41.5 [31.3; 48.6]	37.0 [32.6; 41.4]	0.21	51.8 [41.3; 55.4]	37.3 [35.2; 40.3]	0.22
Vit. B5 (mg)	6.75 [5.65; 11.5]	5.50 [4.57; 6.07]	0.23	11.4 [6.98; 12.5]	5.40 [4.85; 5.57]	0.20
Vit. B6 (mg)	2.45 [2.10; 3.68]	2.10 [1.90; 3.18]	0.33	3.50 [2.32; 4.15]	2.40 [1.88; 3.00]	0.30
Biotin (µg)	47.5 [25.8; 85.8]	32.0 [19.8; 44.5]	0.24	77.6 [44.4; 86.2]	30.6 [26.2; 33.6]	0.14
Vit. B9 (µg)	320 [284; 486]	282 [258; 310]	0.14	524 [442; 602]	346 [237; 499]	0.30
Vit. B12 (µg)	6.40 [4.12; 9.00]	4.85 [4.32; 6.23]	0.34	7.20 [6.40; 8.20]	5.55 [3.57; 7.60]	0.17
Vit. C (mg)	162 [97.2; 229]	138 [80.6; 191]	0.61	186 [137; 273]	130 [104; 144]	0.28
Vit. A (µg)	1076 [806; 1646]	822 [489; 1172]	0.13	1788 [784; 2100]	1024 [718; 1200]	0.08
Vit. D (µg)	4.25 [1.50; 6.98]	1.45 [0.87; 3.30]	0.12	6.55 [2.75; 8.90]	3.10 [2.33; 3.35]	0.22
Vit. E (mg)	11.3 [7.57; 17.7]	9.15 [6.90; 11.2]	0.59	18.3 [14.7; 20.4]	8.35 [6.95; 9.75]	0.035
Vit. K (µg)	113 [90.1; 174]	98.0 [59.1; 146]	0.37	87.0 [68.3; 150]	151 [95.3; 274]	0.28

Figure S2. Correlogram between maternal body composition, and breast milk hormones. Data show Spearman (Rho) coefficient: p-values lower than 0.05 are shown in color (red positive correlations, blue negative correlations); otherwise, the grid is blank. BMI: body mass index; WHI: waist-to-hip index; GIP: gastrointestinal peptide.

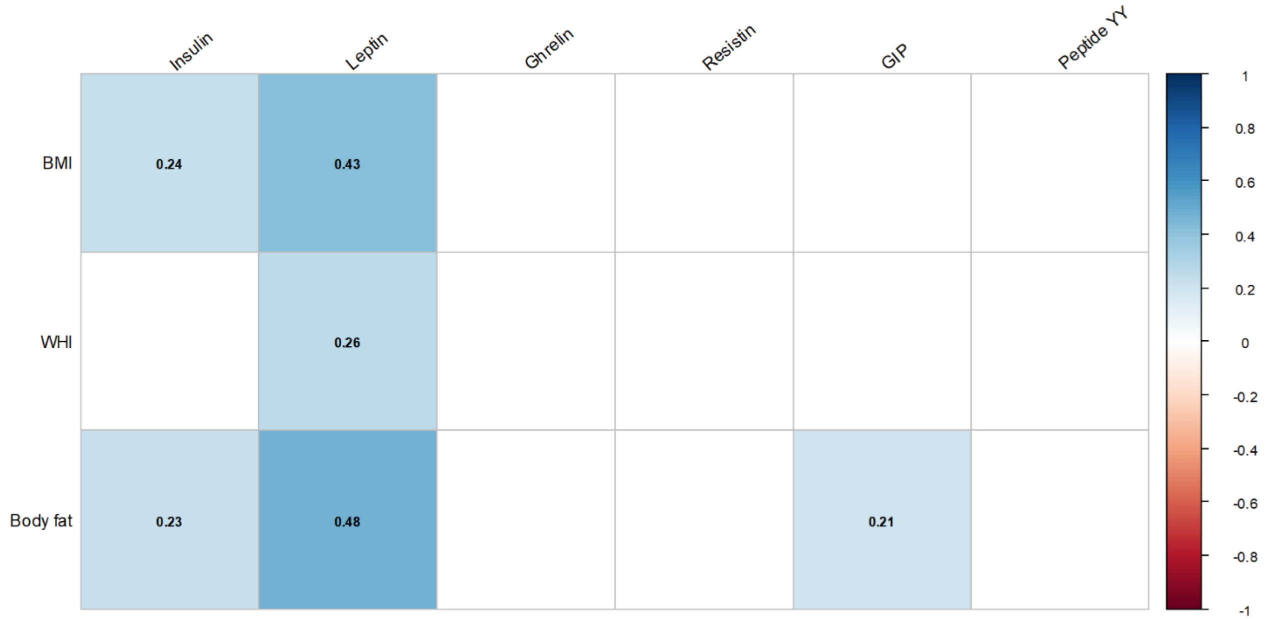

Table S2. Linear mixed models assessing the effect of antenatal corticosteroids and magnesium sulfate in women with preterm labor on breast milk hormones and their influence on infants' weight, length, and head circumference (head circ.). Women by day were included as random effect. Data show beta ± standard error (SE) and the associated P-value (P).

		**Insulin**	**Leptin**	**Ghrelin**	**Resistin**	**GIP**	**Peptide YY**
**Weight**	Corticosteroids	-0.51±0.21 (P=0.031)	-0.59±0.21 (P=0.010)	-0.62±0.22 (P=0.011)	-0.55±0.19 (P=0.011)	-0.44±0.22 (P=0.06)	-0.49±0.21 (P=0.031)
	Magnesium sulfate	-0.11±0.23 (P=0.64)	-0.10±0.22 (P=0.65)	-0.22±0.21 (P=0.33)	-0.23±0.19 (P=0.24)	-0.40±0.24 (P=0.11)	-0.29±0.21 (P=0.19)
**Length**	Corticosteroids	-0.14±0.07 (P=0.06)	-0.16±0.07 (P=0.031)	-0.18±0.07 (P=0.031)	-0.15±0.07 (P=0.040)	-0.12±0.07 (P=0.11)	-0.13±0.08 (P=0.11)
	Magnesium sulfate	-0.07±0.07 (P=0.38)	-0.08±0.07 (P=0.29)	-0.10±0.07 (P=0.17)	-0.10±0.07 (P=0.17)	-0.16±0.08 (P=0.06)	-0.12±0.07 (P=0.10)
**Head circ.**	Corticosteroids	-0.09±0.07 (P=0.20)	-0.11±0.06 (P=0.12)	-0.11±0.07 (P=0.13)	-0.10±0.07 (P=0.15)	-0.06±0.07 (P=0.39)	-0.06±0.07 (P=0.41)
	Magnesium sulfate	-0.11±0.07 (P=0.15)	-0.09±0.07 (P=0.21)	-0.13±0.07 (P=0.07)	-0.13±0.07 (P=0.07)	-0.20±0.07 (P=0.011)	-0.16±0.07 (P=0.021)

Figure S3. Linear mixed models on the association between maternal anthropometry (A) and maternal dietary pattern (B) on breast milk hormones that did not influence neonatal growth in each cohort. Women by day were included as random effect and maternal variables were normalized by logarithmic transformation. Data show beta and standard error (SE). Red lines mean significant association ($P<0.05$), and non-significant associations are represented in black. BMI: body mass index; WHI: waist-to-hip index; SFAs: saturated fatty acids; MUFAs: monounsaturated fatty acids; PUFAs: polyunsaturated fatty acids.

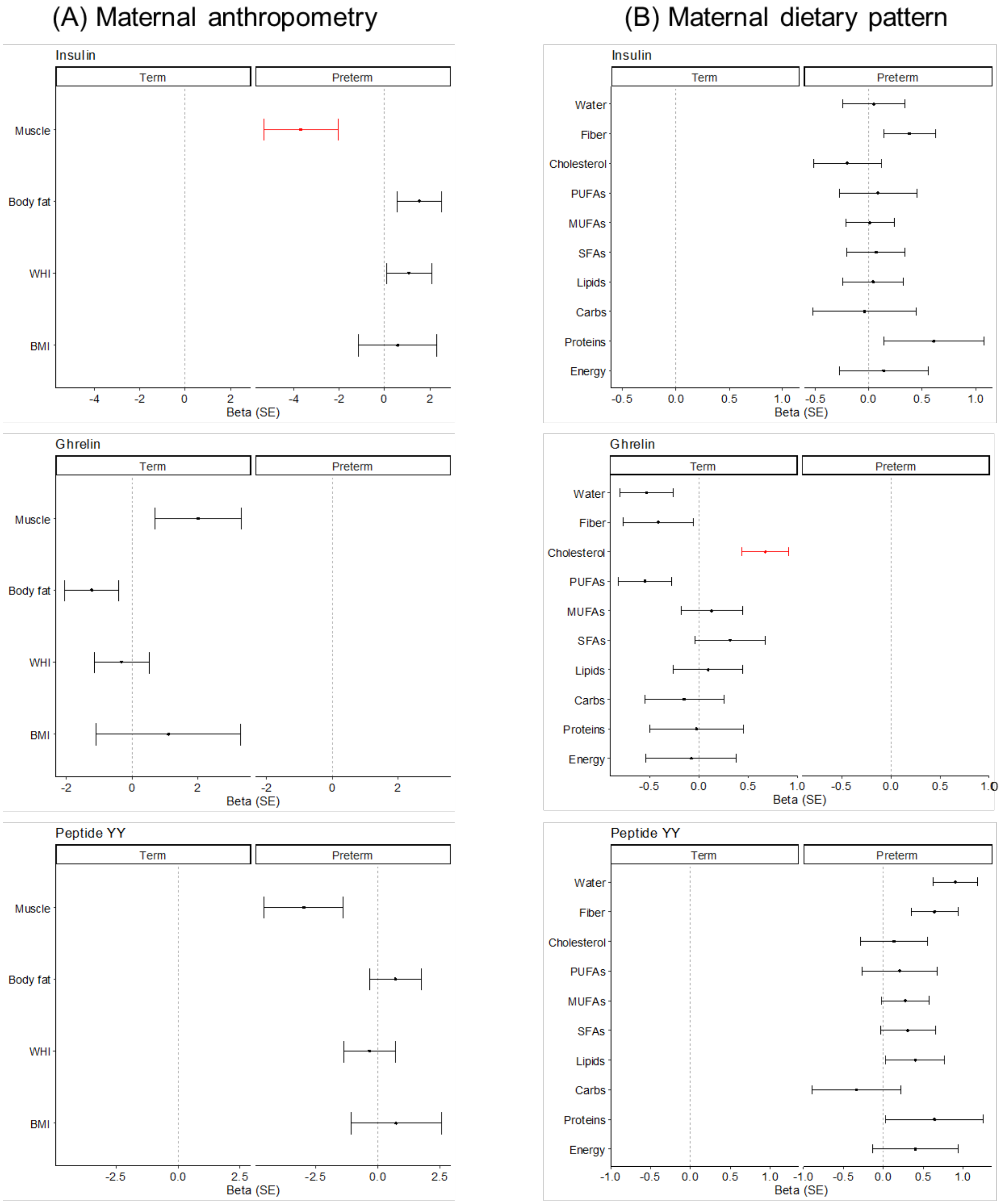

Supplement: Supplementary file 1 [file DataSheet_1.pdf]
